# Measurement of Feline Alpha-1 Acid Glycoprotein in Serum and Effusion Using an ELISA Method: Analytical Validation and Diagnostic Role for Feline Infectious Peritonitis

**DOI:** 10.3390/pathogens13040289

**Published:** 2024-03-29

**Authors:** Pierpaolo Romanelli, Walter Bertazzolo, Andrea Prisciandaro, Andrea Leone, Ugo Bonfanti, Saverio Paltrinieri

**Affiliations:** 1 MYLAV Veterinary Laboratory, 20017 Passirana di Rho, Italy; pierpaoloromanelli@mylav.net (P.R.); andreaprisciandaro@mylav.net (A.P.); andrealeone@mylav.net (A.L.); ugobonfanti@laboratoriolavallonea.it (U.B.); 2 Department of Veterinary Medicine and Animal Sciences, University of Milan, 26900 Lodi, Italy; saverio.paltrinieri@unimi.it

**Keywords:** AGP, accuracy, acute-phase proteins, FIP, likelihood ratio, specificity, sensitivity, validation

## Abstract

Background: Alpha-1 acid glycoprotein (AGP) may support a clinical diagnosis of feline infectious peritonitis (FIP). In this study, we assessed the analytical and diagnostic performances of a novel ELISA method to measure feline AGP. Methods: AGP was measured in sera and effusions from cats with FIP (n = 20) or with other diseases (n = 15). Precision was calculated based on the coefficient of variation (CV) of repeated testing, and accuracy was calculated by linearity under dilution (LUD). Results: The test is precise (intra-assay CVs: <6.0% in individual samples, <15.0% in pooled samples; inter-assay CVs <11.0% and <15.0%) and accurate (serum LUD r^2^: 0.995; effusion LUD r^2^: 0.950) in serum and in effusions. AGP is higher in cats with FIP than in other cats in both serum (median: 1968, I-III interquartile range: 1216–3371 μg/mL and 296, 246–1963 μg/mL; *p* = 0.009) and effusion (1717, 1011–2379 μg/mL and 233, 165–566 μg/mL; *p* < 0.001). AGP discriminates FIP from other diseases (area under the receiver operating characteristic curve: serum, 0.760; effusion, 0.877), and its likelihood ratio is high (serum: 8.50 if AGP > 1590 μg/mL; effusion: 3.75 if AGP > 3780 μg/mL). Conclusion: This ELISA method is precise and accurate. AGP in serum and in effusions is a useful diagnostic marker for FIP.

## 1. Introduction

Feline infectious peritonitis (FIP) is a systemic disease caused by the feline coronavirus (FCoV), which is particularly frequent in young cats living in multi-cat environments. If untreated, FIP is invariably fatal. Recently, antiviral treatments have been established and demonstrated to be effective [1,2,3], although a few side effects have been reported [4,5,6,7] and very little information is available about possible long-term effects [7,8]. Treatment is recommended where the drugs is legally available [3], but unfortunately, in the large majority of countries, the drug is not licensed; therefore, treatments are not allowed. Therefore, an accurate diagnosis is mandatory either to treat the cats or to decide on euthanasia. The diagnosis of dry FIP may be challenging because only the demonstration of typical lesions by histology, possibly followed by the detection of intralesional FCoV antigens by immunohistochemistry or viral RNA by PCR, can confirm the disease. On the contrary, the diagnosis of FIP in the wet forms may be supported by several findings, such as the typical macroscopic appearance of the fluid; high specific gravity; high protein content; a positive Rivalta test; the presence of a non-specific inflammatory cell population with a proteinaceous background; and, more accurately, by a high ratio between the activity of the lactate dehydrogenase enzyme and the total nucleated cell count (LDH/TNCC ratio) or by a high delta-Total Nucleated Cells (TNC) measured with a Sysmex XT-2000iV laser-based automated cell counter [9,10,11,12,13]. Additionally, in both dry and wet forms, the disease may be highly suspected if routine bloodwork evidences anemia; microcytosis; lymphopenia; and, in particular, hyperproteinemia, hypoalbuminemia, hyperglobulinemia, and increased α_2_ and γ-globulin with polyclonal appearance in serum protein electrophoresis [12,14,15]. The identification of FCoV by PCR in effusion may confirm the disease, but the test has a sub-optimal sensitivity, and some cats with FIP may be negative [11,12,16,17,18,19,20,21].

Serum alpha-1 acid glycoprotein (AGP) is another important marker for FIP. As an acute-phase protein, AGP may increase in any inflammatory condition, but it has been demonstrated that increases in AGP are more frequent and more severe in the serum of cats with FIP than in serum from cats with other disease [22,23,24]. Therefore, an increased serum concentration of AGP is considered an accurate diagnostic tool for FIP [24,25,26,27]. A few reports have also described the possible utility of AGP measurement in the effusions [22,24,28,29]. With the exception of a study that employed an immunoturbidimetric method [28], most of studies supporting a diagnostic role of AGP for the diagnosis of FIP were conducted using a radial immunodiffusion kit specific for feline AGP [22,23,24,25,26,27]. Recently, ELISA methods have been developed to measure AGP in feline serum. One of these methods has been validated for use in serum [30], while the other has not yet been validated, although it has been used in cats with FIP or other disorders [31,32], demonstrating an important role as a predictor of recovery after antiviral treatment [31].

The aim of this study was to determine the ability of this novel ELISA assay to support a clinical diagnosis of FIP when applied to serum and effusions of cats with and without this condition after a preliminary evaluation of the precision and accuracy of the tests on both biological fluids.

## 2. Materials and Methods

### 2.1. AGP Measurement

AGP was measured using a commercially available ELISA kit (Nextmune Ltd., Wetherby, UK) already used in previous studies [31,32], following the manufacturer’s instructions. Briefly, 100 µL of diluted serum or effusion samples, control material provided by the manufacturer (AGP concentrations: low level, 500 μg/mL; high level, 3000 μg/mL), and standards were added to wells coated with anti-feline AGP antibody. After incubation (60 min at room temperature) and three washes with the wash buffer included in the kit, the captured AGP was detected using an anti-feline AGP antibody conjugated with horseradish peroxidase (HRP). Following incubation (60 min at room temperature), the plate was washed six times; then, 100 µL of 3,3′,5,5′-tetramethylbenzidine (TMB) substrate was added. After allowing the reaction to proceed for 13 min, it was stopped with stop solution (sulphuric Acid 0.5 M), resulting in a yellow color whose intensity is proportional to the amount of AGP in the sample, which can be measured at a wavelength of 450 nm. The concentration of AGP in the sample was then determined by comparing the results of serum or effusion samples with those of standards containing known concentrations of AGP and multiplying the value obtained in the samples by the appropriate dilution factor.

### 2.2. Intra-Assay Precision

The intra-assay precision was assessed first on 10 serum samples and in 10 effusion samples randomly selected from the routine activity of the diagnostic laboratory, irrespective of the presence of other hematological or biochemical abnormalities, that were processed and read in duplicate in the same ELISA plate.

Then, pooled samples were formed with the 4 sera or the 5 effusions with the highest AGP concentration (high intra-assay pool) or with the 4 sera or the 5 effusions with the lowest AGP concentration (low intra-assay pool). Five wells of the same plate were filled with each pool.

### 2.3. Inter-Assay Precision

The inter-assay precision was assessed on 25 serum samples and in 25 effusion samples randomly selected from the routine caseload of the laboratory in order to cover a wide range of AGP concentrations. These samples were processed within 4 h of arrival in the lab; then, they were frozen at −20 °C for a maximum of 4 months, thawed, and processed again in a new plate.

As for the intra-assay testing, the inter-assay precision was also assessed on pooled samples formed using 20 sera or 5 effusions with high AGP concentrations (high inter-assay pool) and 20 sera or 5 effusions with low AGP concentrations (low inter-assay pool) that were frozen just after the formation of the pool, thawed, and read in 5 sequential work sessions using different plates.

### 2.4. Accuracy Determination by Linearity under Dilution (LUD)

Either in serum or in the effusion, the LUD test was performed using the high pool (HP) used for intra-assay precision testing, on which AGP was measured after dilution with deionized water (DW) as follows: 100% (undiluted HP), 80% (HP:DW = 4:1 *v*:*v*), 60% (HP:DW = 3:2 *v*:*v*), 40% (HP:DW = 2:3 *v*:*v*), 20% (HP:DW = 1:4 *v*:*v*), or 0% (DW alone). 

### 2.5. Comparison of Results Obtained in Cats with and without FIP

This part of the study was performed on 35 paired effusion and serum samples, each collected from the same cat on the same day of sampling, and submitted to the laboratory during the routine diagnostic activity. The only inclusion criterion was the availability of data from routine testing about the following:

- Routine hematology performed using an ADVIA 2120 (Siemens Healthineers, Dublin, Ireland);
- Complete clinical chemistry panel run on a Beckman AU5800 (Beckman Coulter, Tokyo, Japan) with special emphasis on the concentrations of total protein, albumin, creatinine, and urea;
- Serum protein electrophoresis performed on a Capillarys 2 (Sebia, France);
- Physicochemical analysis of the effusion, including the macroscopic appearance of the fluid, refractometric estimation of specific gravity, and total nucleated cell count (TNCC) obtained with an ADVIA 2120 counter, as well as spectrophotometric measurement of total bilirubin (3,5-dichlorophenyldiazonium tetrafluoroborate method), cholesterol (esterase/peroxidase method), triglycerides (glycerol phosphate oxidase method), urea (urease method), creatinine (Jaffe reaction), total protein (biuret method), albumin (bromocresol green method), and LDH (enzymatic–kinetic method) using the Beckman instrument mentioned above and reagents provided by the manufacturer of the instrument;
- Cytology of the effusion determined through microscopic analysis of smeared and cytocentrifuged specimens stained with May Grunwald-Giemsa and analyzed by board-certified clinical pathologists;
- After RNA extraction using the QIAsymphony^®^ DSP Virus/Pathogen Mini Kit (Qiagen S.p.A., Milan, Italy), real-time PCR (Reliance One-Step Multiplex RT-qPCR Supermix, Bio-Rad Laboratories Srl, Segrate, Milan, Italy) was performed to detect feline coronavirus RNA using the primers and amplification protocol previously described in the literature [33].

Using the data generated with the diagnostic approach, samples were grouped as follows:

- FIP: 20 cases on which all the following changes were present: hyperproteinemia, hypoalbuminemia, inverted A/G ratio, serum protein electrophoresis characterized by increased α_2_ and γ-globulin (polyclonal peak), effusions macroscopically consistent with FIP (yellowish and thick, possibly with fibrin clots) and characterized by high SG (>1.015), high protein content (>20 g/L), albumin:globulin ratio <1.0, low cellularity (<5 × 10^9^/L), high LDH/TNCC ratio (>0.62), cytology consistent with FIP (non-degenerated neutrophils, absence of bacteria, low numbers of macrophages and lymphocytes, and presence of a proteinaceous granular background), and positive RT-PCR for FCoV [11,13,14,15,16].
- Not FIP: 15 cases on which macroscopical, physicochemical, and cytological analyses of the fluid were not consistent with FIP; RT-PCR for FCoV was negative; and cytology revealed the presence of a disease other than FIP (e.g., neoplastic, septic, or lymphocyte-rich effusions/chylous effusions), possibly associated with chemical findings suggestive of another condition (e.g., low protein content or high triglyceride content).

The statistical adequacy of this caseload for the purpose of this study was assessed through a power analysis that demonstrated that the minimum database to achieve reliable results with a probability of α-error <5%, and a power >80% when comparing 2 populations with an enrollment ratio of about 1.3 (20/15 as in this case) and assuming that AGP values in the FIP group can be at least 3 times higher than in the other group [24] should include at least 32 samples (18 with and 14 without FIP).

### 2.6. Statistical Analysis

Statistical analysis was performed using Analyse-it software for Microsoft Excel (Analyse-it Software Ltd., Leeds, UK).

Results obtained in the repeated analysis of the same sera (or pool of sera) employed in intra- and inter-assay precision testing were used to calculate the mean value and the standard deviation; the coefficient of variation (CV) was calculated using the following formula: CV = DS/mean × 100.

The correlation between the percentage of recovery and the expected values of the LUD test, calculated on the basis of the AGP concentration of undiluted pools, was assessed using a least squares regression test. The acceptability of the analytical performance was evaluated using published data on the performance of other assays (state of the art) as a benchmark, as recommended when other methods to assess the clinical demand of acceptability are not available [34].

The concentration of AGP recorded in sera or in effusions of cats with FIP, as well as the AGP effusion:serum ratio recorded in cats with FIP, was compared with those of the “not FIP” group using a non-parametric *t*-test for independent samples (Mann–Whitney U-test). 

In order to assess the discriminating power of AGP in serum and in effusion or of the AGP effusion:serum ratio to support a diagnosis of FIP, for each operating point (e.g., each point value recorded in the study population), we calculated the number of true positives, false positives, false negatives, and true negatives, which are defined as follows:

- True positives: samples from cats with FIP that had values higher than the operating point;
- False positives: samples from cats without FIP that had values higher than the operating point;
- False negatives: samples from cats with FIP that had values lower than the operating point;
- True negatives: samples from cats without FIP that had values lower than the operating point.

For each operating point, the sensitivity (Sens), specificity (Spec), and positive likelihood ratio (LR+) were calculated using standard formulae [35]. Then, a receiver operating characteristic curve (ROC curve) was generated by plotting Sens vs. 1-Spec, and the area under the curve (AUC) was calculated. The AUC values were then compared to each other, and cut-off values with equal sensitivity and specificity, as well as those with the highest specificity or positive likelihood ratio, were then calculated [35]. 

## 3. Results

### 3.1. Precision and Accuracy of AGP Measurement

Results regarding intra- and inter-assay precision are reported in Table 1. In duplicate analyses of individual serum samples, the intra-assay CVs were lower than 10%, except for one sample (CV, 10.8%; AGP, 269.9 μg/mL) while the inter-assay CVs were lower than 15%, although in four samples with AGP concentrations ranging from 2023.5 to 3597.8, the CV was higher than 20%. The intra- and inter-assay CVs of pooled sera were slightly higher, especially for the low pool, but always lower than 15%. 

In the effusions, the median CVs of duplicate analysis of individual samples were similar to those of serum samples; only two out of the 10 samples included in the intra-assay testing (AGP concentrations: 2397.1 and 1464.5 μg/mL) had a CV higher than 10% (11.1% and 11.3%, respectively). Similarly, only 2 out of the 25 samples included in the inter-assay testing, both with a very low AGP concentration (87.0 and 88.3 μg/mL) had a CV higher than 15%, but although in one of these two, cases the CV was only slightly higher than this threshold (15.9%), in the other, the CV was very high (56.5%). As for serum, the intra-assay precision of pooled samples was slightly lower than that of individual samples, although the magnitude of the CVs of pooled effusions was similar to that of pooled sera.

**Figure 1:**
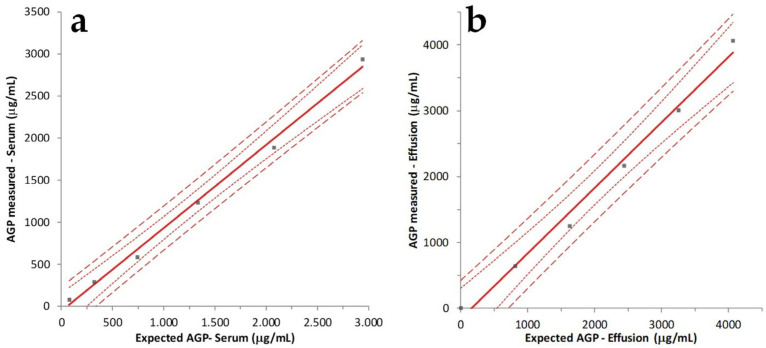
Linearity under dilution recorded in serum (**a**) and effusion (**b**). The solid red lines indicate the linear fit, the dotted lines represent the 95% confidence interval (CI), and the dashed lines represent the 95% prediction interval.

The correlation between expected and obtained values in the LUD test was excellent in sera (*p* < 0.001; r^2^ = 0.995) and in effusions (*p* = 0.001; r^2^ = 0.990) (Figure 1).

### 3.2. Group Comparison

According to the inclusion criteria, all the FIP cases were considered as affected by FIP. The “not FIP” group included six cases with a neoplastic effusion (three lymphoid, two epithelial, and one of unknown origin), four chylous effusions, three septic effusions, one hemorrhagic effusion, and one lymphocyte-rich transudate likely associated with cardiomyopathy [36].

**Figure 2:**
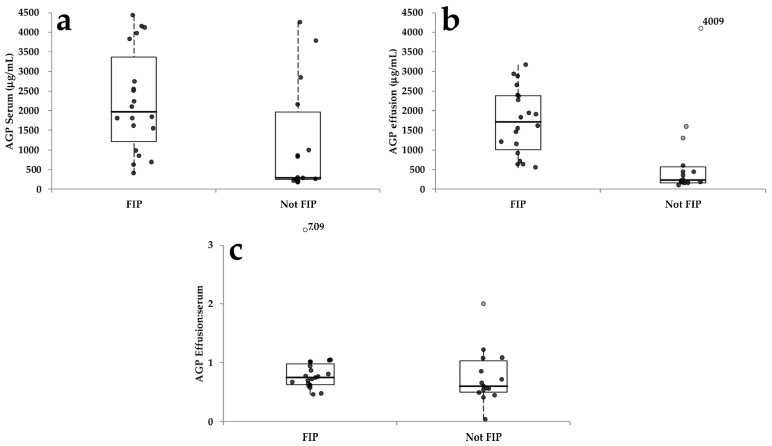
AGP concentration in serum (**a**) and effusion (**b**) and AGP effusion:serum ratio (**c**) in cats with FIP or with other diseases. The boxes indicate the I–III interquartile range (IQR), the horizontal lines indicate the median value, whiskers extend to further observation within the 1st quartile minus 1.5 × IQR or to further observation within the 3rd quartile plus 1.5 × IQR. Black dots indicate values not classified as outliers, gray dots indicate near-outliers (values exceeding the third quartile ± (1.5 × IQR)), and white dots indicate far outliers (values exceeding the third quartile ± (3.0 × IQR)). In order to expand the figures, the scale regarding the ratio was limited to 3, and one far outlier that exceeded the limit of the scale is reported on the top of the graph, along with the corresponding value. The value recorded for the far outlier detected in the “Not FIP” group is also included in the graph.

Results regarding the concentration of AGP in sera and effusions and the AGP effusion:serum ratio are reported in Table 2 and summarized in Figure 2. As shown in the table, the concentration of AGP was higher in cats with FIP than in cats with other diseases in both sera and effusions. Conversely, no differences in the AGP effusion:serum ratio were found between the two groups.

However, results regarding the “not FIP” group were very dispersed, especially in serum, due to the heterogeneous composition of this group in terms of types of diseases (Table 3). 

Although a statistical comparison was not possible due to the low number of samples per group, the concentration of AGP was low in the serum of cats with chylous effusions and lymphocyte-rich transudates and high in the serum of cats with hemorrhagic, septic, and neoplastic effusions. Conversely, the concentration of AGP in the effusions was low to moderate in all the groups except in septic effusions. As a consequence, the ratio was generally lower than 1, with the exception of the only lymphocyte-rich transudate. No apparent relationship was found between the cell counts and the AGP concentration of the effusions.

**Figure 3:**
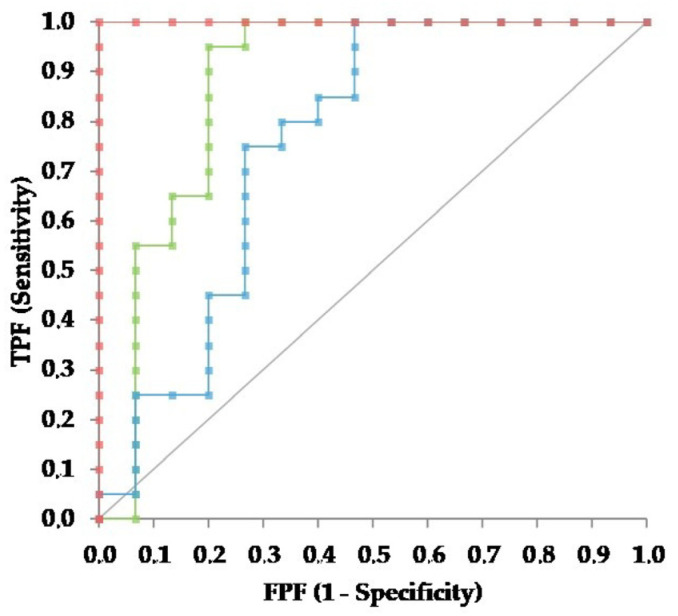
Receiver operating characteristic curves regarding the AGP in sera (light blue) and effusions (green) and the AGP effusion:serum ratio (red). The gray line is the no-discrimination line.

The ROC curves designed using the data reported above (Figure 3) demonstrate that the concentrations of AGP in sera and effusions were significantly different from the line of discrimination (*p* < 0.001 for both), while the effusion:serum ratio was not (*p* = 0.286). The area under the ROC curve (AUC) regarding the concentrations of AGP in effusions (0.877; 95% CI: 0.732–1.022) was significantly higher than the AUC of the effusion:serum ratio (0.617; 95% CI: 0.402–0.831) (*p* = 0.026), while no significant differences were found between the AUC regarding the concentrations in effusions and the AUC regarding the concentrations in sera (0.760; 95% CI: 0.579–0.941) (*p* = 0.114) nor between the AUC regarding the concentrations in sera and the AUC regarding the effusion:serum ratio (*p* = 0.379).

The diagnostic performances calculated on the basis of the ROC curves for the concentrations of AGP in sera and effusions are reported in Table 4.

The results reported in Table 4 evidence that, in both sera and effusions, absolute sensitivity and specificity correspond to very low and very high AGP values, respectively. Similarly, the highest likelihood ratio is achieved only at very high APG values. Conversely, the AGP concentrations that maximize sensitivity and specificity (around 80% in the effusion and 75% in serum) or that are characterized by the highest Youden indexes are notably lower than 1500 μg/mL, the threshold value recommended to support a clinical diagnosis of FIP with other methods [22,25]. As expected from the AUC of the ROC curve, the diagnostic performances of the AGP serum:effusion ratio are not satisfactory.

## 4. Discussion

The ELISA method used in the current study is sufficiently precise and accurate to be used in practice to differentiate the serum concentration of AGP in cats affected by diseases on which this analyte is expected to increase. More specifically, the CVs obtained by repeated reading of individual samples were generally lower than 10% in intra-assay testing or slightly higher than 10% in inter-assay testing, except in samples characterized by low concentrations of AGP, for which the intra-assay precision decreased. The latter finding is not unexpected, since the inverse relationship between mean values and the coefficient of variation is well known [37]. Ultimately, the performances on individual samples were comparable with those recorded in a previous study [30] using a different ELISA kit on pooled samples. Conversely, the CVs recorded in the current study on pooled samples were higher than those recorded in duplicate readings of individual samples, as already reported in studies comparing CVs obtained in individual vs. pooled samples [38]. In any case, the magnitude of precision was similar to those of many analytes routinely used in clinical pathology [39] and to those of other ELISA tests that can be used as a benchmark for the current method [30,40,41]. It is also noteworthy that precision levels were similar in serum and in effusions, despite the fact that the latter fluid may have physicochemical properties such as viscosity, the presence of cellular debris, and variable concentrations of proteins or other plasmatic constituents that are very different from those of serum and that are known to potentially create a matrix effect and interfere with an immunoassay [42].

In the absence of a gold standard to compare the results of the ELISA method, accuracy was determined using a LUD test. This approach demonstrated a high level of correlation between expected and obtained values in both sera and effusions, although in the effusions the correlation coefficient was lower than in sera, likely due to the lower fluidity of effusion sampling, which may have influenced the accuracy of the dilutions. However, the high correlation coefficients recorded in both the specimens suggest that the accuracy of the method is also sufficient to use this test in routine diagnostic practice either in serum or in effusion.

The measurement of AGP in sera and effusions of cats with FIP and with other diseases confirmed that using the method employed in the current study, this acute-phase protein may support a clinical diagnosis of FIP, as already demonstrated using previous methods [22,23,24,25,26,27], since the values of the FIP group were largely higher than those of the other groups, and the magnitude of this difference was higher than the analytical intrinsic imprecision of the method. In serum, the threshold considered as definitely confirmatory of FIP in the current study was notably higher than that identified years ago as highly suggestive of FIP using the radial immunodiffusion method (1500 μg/mL) and also used in recent studies based on the same method employed in the current study [22,31]. This likely depends on the presence of cats with septic effusions in the “not FIP” group of the current study, in which the AGP is expected to be high due to its role as a positive acute-phase protein [27]. However, although the threshold needed to obtain the absolute specificity or the maximum LR+ for FIP is very high, the best combination of sensitivity and specificity (approximately 75% each, accounting for an LR+ close to 3) is achieved at AGP concentrations notably lower than the previous threshold. Therefore, although the results of samples from cats with FIP were compared with those of cats with diseases characterized by an inflammatory pathogenesis that induce an increase in the serum concentration of acute-phase proteins, this study confirms that measurement of serum AGP is a powerful tool to support a clinical diagnosis of FIP when the pre-test probability of FIP is high [25].

Compared with the concentrations in sera, the concentrations of AGP in the effusions were slightly lower in all the groups, with a few individual exceptions. This suggests that AGP simply flows from blood to effusion, regardless of the underlying disease. Based on these results, it seems unlikely that AGP accumulates in effusion and, therefore, increases its concentration, as demonstrated for other analytes that can be found in effusions in different diseases [29,43], and that AGP is produced locally by mesothelial, endothelial, or inflammatory cells or by intracavitary lymphoid cells, as demonstrated in other species and/or for other APPs [44,45,46,47,48]. Rather, the slightly lower AGP concentration in effusion compared with serum suggests that, once extravasated in the effusion, AGP is slightly diluted by the additional amounts of fluids that flow into the cavity. It should be stressed that the group of cats with effusions due to diseases other than FIP was quite heterogeneous, and some subgroups were composed by a single case; therefore, it cannot be ruled out that, by increasing this caseload, some peculiar pattern may be found in cats from the “not FIP” group, allowing for the use of the serum:effusion ratio to differentiate FIP from some other conditions, which seems not to be the case with the current caseload. 

However, regardless of the possible mechanisms responsible for the presence of AGP in effusions that, with the design of this study, can only be argued as hypothesized above, the analysis of AGP values evidenced a pattern of distribution of AGP very similar to that recorded in serum, namely that values in cats with FIP were higher than in the other group. Therefore, in effusions, the measurement of AGP may also help to discriminate cats with FIP from cats with other diseases, and the analysis of the ROC curves demonstrated that this discriminating power is even higher than in serum. Therefore, when effusive FIP is suspected, it would be advisable to measure AGP in effusion rather than in serum or, to obtain more complete diagnostic information, to measure AGP in both sera and in effusions. This finding is in agreement with what was previously reported using the single radial immunodiffusion (SRID) kit already validated in cats [24]. Additionally, at the cut-off characterized by the highest Youden index, AGP is very sensitive; therefore, it may work as an exclusion test either in serum or in effusions, allowing FIP to be ruled out when the AGP concentration is below the corresponding thresholds. Therefore, the measurement of AGP may be recommended not only in sera but also in effusions, where other findings such as the detection of neoplastic cells or cytological findings consistent with a septic effusion (which also had high AGP values due to the role of this protein as an acute-phase reactant) may also drive the diagnosis and rule out the diagnosis of FIP. From this standpoint, the fact that the high likelihood ratio recorded in effusions despite the caseload was biased by the inclusion of inflammatory conditions is particularly interesting. According to the Bayesian approach [49], if the pre-test the probability of FIP is low, as in the example of septic effusions described above, the diagnosis of FIP remains unlikely, even in the presence of high AGP values. Conversely, AGP may be used as a confirmatory test when other findings consistent with FIP are present (e.g., yellowish and sticky, gross appearance of the fluid; high protein content; high LDH:TNCC ratio; and non-specific inflammatory cytology) [3,12,13] or may act as a discriminating marker for FIP when some but not all of the findings listed above are present and, therefore, the pre-test probability is intermediate. Conversely, independent of the clinical scenario, low AGP values in sera or effusions may allow FIP to be ruled out.

## 5. Conclusions

In conclusion, the ELISA method used in this study is sufficiently precise and accurate to be used for routine diagnostics in either serum or effusion. Additionally, our results further support the diagnostic importance of the measurement of AGP in serum samples of cats with a clinical suspicion of FIP and evidence that the possible diagnostic role of AGP measurement for FIP is even more important in effusions. Therefore, in routine practice, it may be advisable to measure AGP in effusions to further improve the possibility of correctly diagnosing FIP.

## Figures and Tables

**Table 1:** Results of intra- and inter-assay testing calculated on duplicate readings individual serum (n = 10) or effusion (n = 25) samples or on five readings of pooled sera or effusions with low and high AGP concentrations. The AGP concentrations of duplicate readings of individual samples are reported as minimum and maximum values of the 10 samples included in the intra-assay precision testing or of the 25 samples included in the inter-assay precision testing. The CVs of duplicate readings are reported as median and, between parenthesis, I and III interquartile range.

Specimen	Sample	Intra-Assay		Inter-Assay	
		AGP (μg/mL)	CV	AGP (μg/mL)	CV
Serum	Duplicate samples	151.7–4244.4	5.4% (3.2–7.8%)	226.9–4474.2	10.1% (6.4–14.6%)
	Low pool	180.1	13.5%	225.4	10.2%
	High pool	2727.7	8.6%	2865.4	13.4%
Effusion	Duplicate samples	96.7–4316.6	5.9% (3.0–7.1%)	87.0–4207.8	10.6% (3.7–13.9%)
	Low pool	196.3	9.2%	217.2	10.4%
	High pool	3828.5	12.8%	3674.6	9.2%

**Table 2:** Results regarding the concentration of AGP in serum and effusion and the AGP effusion:serum ratio recorded in cats with FIP and with diseases other than FIP. Results are presented as mean ± standard deviation and as median values (between parenthesis), as well as I–III interquartile range and minimum and maximum values (in square brackets).

Group	Serum AGP (μg/mL)	Effusion AGP (μg/mL)	Effusion/Serum AGP
FIP (n = 20)	2239 ± 1283 (1968) 1216–3371 (405–4428)	1736 ± 832 (1717) 1011–2379 (549–3166)	1.08 ± 1.43 (0.75) 0.63–0.98 (0.46–7.09)
Not FIP (n = 15)	1173 ± 1397 (296) 246–1963 (165–4254)	679 ± 1042 (233) 165–566 (103–4099)	0.75 ± 0.46 (0.60) 0.50–1.03 (0.04–2.00)
	*p* = 0.009	*p* < 0.001	*p* = 0.243

**Table 3:** AGP concentrations in sera and effusions and AGP effusion:serum ratio recorded in cats without FIP. Results are presented as mean ± standard deviation, median values (between parenthesis), I–III interquartile range, and minimum and maximum values (in square brackets).

Group	TNCC × 10³/μL	Serum AGP (μg/mL)	Effusion AGP (μg/mL)	Effusion/Serum AGP
Chylous (n = 4)	14.4 ± 10.7 (9.7) 8.0–22.4 (7.9–30.3)	219 ± 41 (228) 184–252 (165–257)	168 ± 45 (180) 134–198 (103–209)	0.78 ± 0.24 (0.78) 0.58–0.98 (0.49–1.07)
Hemorrhagic (n = 1)	2.0	990	434	0.44
LRT (n = 1)	0.8	192	233	1.21
Neoplastic (n = 6)	238.3–499.1 (41.1) 17.6–170.1 (1.3–1255.9)	1127 ± 1557 (560) 277–1142 (249–4254)	310 ± 182 (257) 159–451 (156–592)	0.70 ± 0.67 (0.54) 0.38–0.76 (0.04–2.00)
Septic (n = 3)	322.8 ± 41.0 (344.4) 286.9–347.7 (275.4–348.4)	2926 ± 815 (2840) 2272–3624 (2158–3780)	2329 ± 1540 (1590) 1347–3681 (1299–4099)	0.75 ± 0.29(0.60) 0.57–1.00 (0.56–1.08)

TNCC = total nucleated cell count; LRT = lymphocyte-rich transudate.

**Table 4:** Results regarding the concentration of AGP in serum and in effusion and the AGP effusion:serum ratio recorded in subgroups of cats without FIP. Results are presented as mean ± standard deviation and as median values (between parenthesis), as well as I–III interquartile range and minimum and maximum values (in square brackets).

Diagnostic Indicator	Serum AGP	Effusion AGP	Effusion/Serum AGP
100%Se	<438 μg/mL	<296 μg/mL	<0.44
100% Sp	>4099 μg/mL	>4254 μg/mL	>2.00
Maximum LR+	8.50 (>1590 μg/mL)	3.75 (>3780.3 μg/mL)	1.80 (>0.71)
Optimal Se/Sp	707 μg/mL (Se 80%; Sp 80%)	990 μg/mL (Se 75%; Sp 73%)	0.69 (Se 60%; Sp 60%)
Max Youden index	0.750 (592 μg/mL) (Se 96%, Sp 80%)	0.533 (296 μg/mL) (Se 100%, Sp 53%)	0.367 (0.56) (Se 90%, Sp 47%)

Se = sensitivity; Sp = specificity; LR+ = positive likelihood ratio.

## Data Availability

The data presented in this study are available upon request from the corresponding author.
